# Patients with dementia: prevalence and type of drug–drug interactions

**DOI:** 10.3389/fphar.2024.1472932

**Published:** 2024-10-28

**Authors:** María Cristina Muñoz-Contreras, Begoña Cerdá, Francisco Javier López-Román, Ignacio Segarra

**Affiliations:** ^1^ Hospital Pharmacy, Hospital La Vega, Murcia, Spain; ^2^ Pharmacokinetics, Patient Care and Translational Bioethics Research Group, Catholic University of Murcia (UCAM), Guadalupe, Spain; ^3^ Department of Pharmacy, Faculty of Pharmacy and Nutrition, Catholic University of Murcia (UCAM), Guadalupe, Spain; ^4^ Nutrition, Oxidative Stress and Bioavailability Research Group, Catholic University of Murcia (UCAM), Guadalupe, Spain; ^5^ Facultad de Medicina, Universidad Católica San Antonio (UCAM), Murcia, Spain; ^6^ Primary Care Research Group, Biomedical Research Institute of Murcia (IMIB-Arrixaca), University Clinical Hospital ‘Virgen de la Arrixaca’, Murcia, Spain

**Keywords:** dementia, Alzheimer’s disease, drug–drug interactions, polymedication, aging, patient Care

## Abstract

**Background:**

Patients with Alzheimer’s disease (AD) and other dementias are more frequently exposed to polymedication, mainly due to the presence of comorbidities, are particularly vulnerable to drug-related problems, and present greater risk of adverse effects due to drug–drug interactions (DDIs).

**Purpose:**

To assess the prevalence of clinically relevant interactions in dementia patients using a routine database, we describe the most frequent interactions and risk factors associated with them to facilitate specific interventions and programs to prevent and minimize them.

**Methods:**

An observational, descriptive, and cross-sectional study that included patients with AD and other types of dementia (n = 100, 64% female) was conducted to identify potential DDI in their treatment using the Lexi-Interact/Lexicomp^®^ database.

**Results:**

A total of 769 drugs were prescribed, involving 190 different active ingredients; 83% of the treatments included five or more drugs. DDI occurred in 87% of the patients, of which 63.2% were female. A total of 689 DDIs were found, grouped in 448 drug pairs, with a mean of 6.9 ± 7.1 (range, 0–31) DDIs per patient, and 680 DDIs were considered clinically relevant. It was observed that 89.8% of the DDIs had a moderate level of severity, 23.5% had a good level of relevance, and pharmacodynamic-based DDIs accounted for 89.5%. The drugs most frequently involved in DDIs were quetiapine (24.5%) and acetylsalicylic acid (10%). A total of 97 DDIs were detected between the acetylcholinesterase inhibitors (AChEIs), and the remaining drugs were administered concomitantly. One of the most frequent DDIs was between AChEIs and beta-blocking agents (n = 29, 4.3%). The most important factors that showed the strongest association with the presence of drug interactions were the use of AChEIs (*p* = 0.01) and the total number of drugs (*p* = 0.014) taken by the patient.

**Conclusion:**

Patients with dementia present increased risk of DDIs. Among the most common drugs are psychotropic drugs, which are involved in pharmacodynamic interactions caused by the concomitant use of CNS-targeted drugs. The results highlight the difficulty to evaluate DDIs in clinical practice due to polymedication and variety of comorbidities. Therefore, it is important to review their treatment and consider metabolism inhibition or induction, and potentially P450 substrate overlapping.

## 1 Introduction

Dementia is a chronic and progressive syndrome; it is characterized by gradual short- and long-term memory loss and behavioral disturbance. Alzheimer’s disease (AD) is presently the most common cause of dementia (60%–70%) (World Health Organization). The progressive aging of the population together with a greater presence of chronic pathologies among the elderly has been directly related to a very significant increase in the consumption of health-care resources, including drugs (Offerhaus, 1997) and specialized caregivers (Muñoz-Contreras et al., 2022).

People with dementia are more frequently exposed to polymedication than those without dementia (Kristensen et al., 2018). A cross-sectional study (Growdon et al., 2021) using the National Ambulatory Medical Care Survey (NAMCS) showed that the number of medications was significantly higher in the elderly with dementia than in those without dementia.

Patients with AD and other types of dementia are mostly elderly with multiple comorbidities (61%) (Doraiswamy et al., 2002) including three or more chronic diseases (Bunn et al., 2014; Bynum et al., 2004) such as hypertension and musculoskeletal, cardiovascular, metabolic/endocrine, and gastrointestinal disorders. Thus, these patients are particularly vulnerable to drug-related problems and have higher risk of adverse effects or their occurrence as a consequence of a drug–drug interaction (DDI) (Doraiswamy et al., 2002; Bunn et al., 2014; Kuo et al., 2008). In addition, age-related physiological changes in the elderly may lead to pharmacokinetic and pharmacodynamic changes as well as increased drug sensitivity (Reeve et al., 2017; Mangoni and Jackson, 2004).

DDIs describe the ability of a drug to modify the action or effects of another drug administered successively or simultaneously, causing the latter to undergo a quantitative or qualitative change (De Cos, 2008). DDIs can be beneficial, but they can also be detrimental to the patient by causing negative effects either by over- or underacting a drug (De Cos, 2008). There are three types of DDIs depending on their mechanism of action: pharmaceutical, pharmacokinetic, and pharmacodynamic interactions. Sometimes, pharmacokinetic and pharmacodynamic mechanisms may converge in the same interaction.

- Pharmaceutical interactions occur when two compounds are physically or chemically incompatible with each other. Physical incompatibility causes changes in turbidity, coloration, or even precipitation, whereas chemical incompatibility causes a loss of activity by degradation or inactivation (Beijnen and Schellens, 2004).
- Pharmacodynamic interactions are those produced by the influence of one compound on the effect of another one on the receptors or effector organs where it acts. In patients with dementia, pharmacodynamic changes usually increase sensitivity to some drugs, especially psychotropic drugs (Mangoni and Jackson, 2004). Antipsychotic drugs influence protein phosphorylation through their ability to inhibit dopaminergic D2 receptors (World Health Organization), which are abundantly expressed in the striatum (the brain area where responses to antipsychotic drug exposure are most notable). This inhibition activates adenylyl cyclase, increases cyclic AMP levels, and activates protein kinase A (PKA) (Offerhaus, 1997), which is responsible for phosphorylating receptors and ion channels in the synapse, as well as modulating synaptic function and the activity of other protein kinases. These drugs, especially the atypical antipsychotics, have a more generalized, although moderate, impact on all neurotransmitter systems and brain regions, undoubtedly affecting neuroplasticity as they induce gene expression in many brain areas. Among them is haloperidol, which mediates the gene expression through a PKA-dependent signal transduction pathway (Muñoz-Contreras et al., 2022). These mechanisms could be related to the neurotoxic effect induced by the interaction between antidepressant and antipsychotic drugs.
- Pharmacokinetic interactions are those produced by modifications of the triggering drug on the processes of absorption, distribution, metabolism, and excretion (ADME) of the other drug whose effect may be modified (De Cos, 2008). Most drugs are metabolized by cytochrome (CYP) P450 enzymes in the liver or in the intestinal wall upon absorption, through phase I metabolism (e.g., oxidation, reduction, and hydrolysis among others), whose capacity declines with age (Reeve et al., 2017; Butler and Begg, 2008; McLachlan et al., 2011; Andres et al., 2019). These enzymes are mostly responsible for metabolizing some of the drugs used by dementia patients, such as neuroleptics, antidepressants, and anxiolytics. Metabolism-based interactions generally occur due to overlapping substrate specificity. Thus co-administration of two or more P450 enzyme (e.g., 2A4 and 2D6 isoforms) substrates would lead to decreased clearance of the drugs, potentially leading to greater effects. In addition, it should also be noted that the blood–brain barrier is altered with age with higher permeability in dementia patients (Farrall and Wardlaw, 2009), which increases the likelihood of enhanced drug penetration across the BBB to reach the CNS (Reeve et al., 2017; Mehta et al., 2015; Nicolazzo and Mehta, 2010). This may further alter the drug response, increasing patients’ sensitivity to their neurological effects (Nicolazzo and Mehta, 2010; Pardridge, 2012; Alajangi et al., 2022). Therefore, elderly patients with dementia are extremely sensitive to the occurrence of medication-related problems, especially DDIs and adverse reactions (ADRs) (Nicolazzo and Mehta, 2010).

About 5% of all hospitalizations in elderly patients result from potentially relevant DDIs (Becker et al., 2007), which occur in almost one-third of elderly patients (Johnell and Klarin, 2007). The presence of dementia is associated with an increased risk of hospitalization (Bynum et al., 2004). In fact, DDIs were the cause of 6.9% of drug-related hospitalizations in older people with dementia or cognitive impairment (Gustafsson et al., 2016) and 17% of ADRs causing hospitalization or problems in the patient’s overall drug therapy (Pirmohamed et al., 2004). Furthermore, several clinical studies with dementia patients have described large variability in the epidemiology of potential DDIs, as well as a high prevalence, ranging from 43.2%–76% (Ruangritchankul et al., 2020; Bogetti-Salazar et al., 2016; Sönnerstam et al., 2018; Oesterhus et al., 2017; Trevisan et al., 2021).

Knowledge and proper management of DDIs can improve the safety and effectiveness of treatments in these patients. However, the studies (Ruangritchankul et al., 2020; Bogetti-Salazar et al., 2016; Sönnerstam et al., 2018; Oesterhus et al., 2017) carried out in this field present disparate methodologies (design, variables collected, and databases), and consequently, the incidence of interactions, their severity, and risk reduction strategies are poorly defined. In addition, the database used in the studies to identify PPIs and DDIs directly affects the results. Several authors have highlighted this heterogeneity between databases, leading to differences in the prevalence of DDIs even in the same study population (Vitry, 2007; Fernández de Palencia Espinosa et al., 2016) as the different databases may use different categories and scales. The Norwegian Medicines Agency scale, which is used to assess the use of psychotropic drugs among outpatients with mild dementia, employs a 3-point scale to classify DDIs into categories of increasing severity: 1-DDIs of academic interest only, 2-DDIs for which physicians should take precautions, and 3-drugs should not be combined because of permanent harm to the patient (Oesterhus et al., 2017). On the other hand, the INTERcheck^®^ Computerized Prescription Support System database, developed by the Mario Negri Institute for Pharmacological Research, uses four severity classes of DDI based on clinical impact: mild, moderate, major, and severe (Trevisan et al., 2021). Similarly, other interactional databases such as Micromedex Drug^®^ and Janusmed^®^ (formerly Sfinx) were used to study the prevalence of DDIs in elderly patients with dementia in a hospital setting (Sönnerstam et al., 2018; Ruangritchankul et al., 2020; Bogetti-Salazar et al., 2016).

This variety of criteria makes it challenging to compare the DDI profile in the daily clinical practice in general, but particularly in patients with Alzheimer’s disease and other dementias also due to their large polymedication context. Therefore, we aim to assess the prevalence of clinically relevant interactions in patients with dementia, using a database of routine use, to describe the most frequent interactions and possible risk factors associated with them to facilitate specific interventions and programs to prevent and minimize them.

## 2 Materials and methods

An observational, cross-sectional, descriptive study was conducted to identify potential DDIs in the treatment of patients with Alzheimer’s disease and other types of dementia. Study data were collected during 2018–2021 in collaboration with the Association of Relatives of People with Neurodegenerative Disease and Prevention of Pathological Aging (AFA Levante, Spain).

The pharmacological treatments were reviewed by means of the electronic prescription and updates of patients’ medical records, and the total number of drugs prescribed was recorded for each treatment. In addition, different types of sociodemographic and clinical variables were included in the study: age, sex, patient’s comorbidities, study level and number, and types of drugs prescribed.

The identification of potential DDI was conducted with the Lexi-Interact/Lexicomp^®^ database which is widely used internationally by health-care professionals (Rodríguez-Terol et al., 2009). This database classifies DDIs according to their level of risk as type A, B, C, D, or X and their level of severity (severe, moderate, and mild) considering severe and moderate levels of clinically relevant DDIs of each pair of drug interactions studied individually.

Qualitative variables were expressed as absolute frequency and their relative frequency in percentages (drug–drug interactions (type and severity), treatment drugs, drug pairs with DDIs, comorbidities, and gender). Continuous variables were evaluated to ensure that they followed a normal distribution and were represented as mean ± standard deviation (SD) (total number of drugs prescribed, total number of comorbidities, and age). The data were processed using SPSS software version 23.0 for Windows^®^. The relationship of the variables under study was analyzed using the Pearson Chi-square test for qualitative variables (association between the existence of IDD and gender and treated with acetylcholinesterase inhibitors (AChEIs)) and the Student’s t-test to analyze quantitative variables with normal distribution (association between the existence of IDD and age, total number of drugs prescribed, and total number of comorbidities).

Finally, the Institutional Ethics Committee of the Catholic University of Murcia reviewed and approved the study (CE041808).

## 3 Results

### 3.1 Patient population features

A total of 100 patients enrolled in the study and their treatments were analyzed. Most of the patients were female (64%) *versus* 36% of male patients, and their mean age were 77.83 ± 10.14 and 81.56 ± 7.41 years for male and female patients, respectively. According to the type of dementia, most patients (61%) had AD, 10% had frontotemporal dementia, 9% had mixed dementia (AD and vascular dementia), 8% had vascular dementia, and 12% were diagnosed with other types of dementia. Because of their clinical status, all of them had a caregiver, male (23%) or female (77%), and it was a first-degree relative in 81% of the patients.

Most patients had multiple pathologies; in fact, 27% had four to five comorbidities and 36% of patients had six or more comorbidities. In addition, 83% of patients were polymedicated, with a mean number of drugs per patient of 7.7 ± 3.3 (range, 2–17). The main comorbidities present in the study population were arterial hypertension and depressive syndrome, with 64% and 61%, respectively (Table 1).

**TABLE 1:** Most frequent comorbidities.

Comorbidity	Value
Arterial hypertension	64%
Depressive syndrome	61%
Dyslipidemia	52%
Ischemic heart disease	32%
Diabetes mellitus II	29%
Hypothyroidism	20%

### 3.2 Pharmacological treatment and drug interaction characteristics

A total of 769 drugs were prescribed, which implied 190 different active pharmacological ingredients. The analysis performed in the Lexi-Interact^®^ database found DDIs in 87% of the patients (63.2% of women and 36.8% men), and 83% of the treatments included five or more drugs. A total of 689 DDIs were found, grouped in 448 drug pairs, with a mean of 6.9 ± 7.1 (range, 0–31) DDIs per patient. Out of them, 680 DDIs were considered clinically relevant, that is, all those with severe and moderate severity levels, regardless of the level of evidence and degree of significance, and were grouped in 441 drug pairs.

**FIGURE 1:**
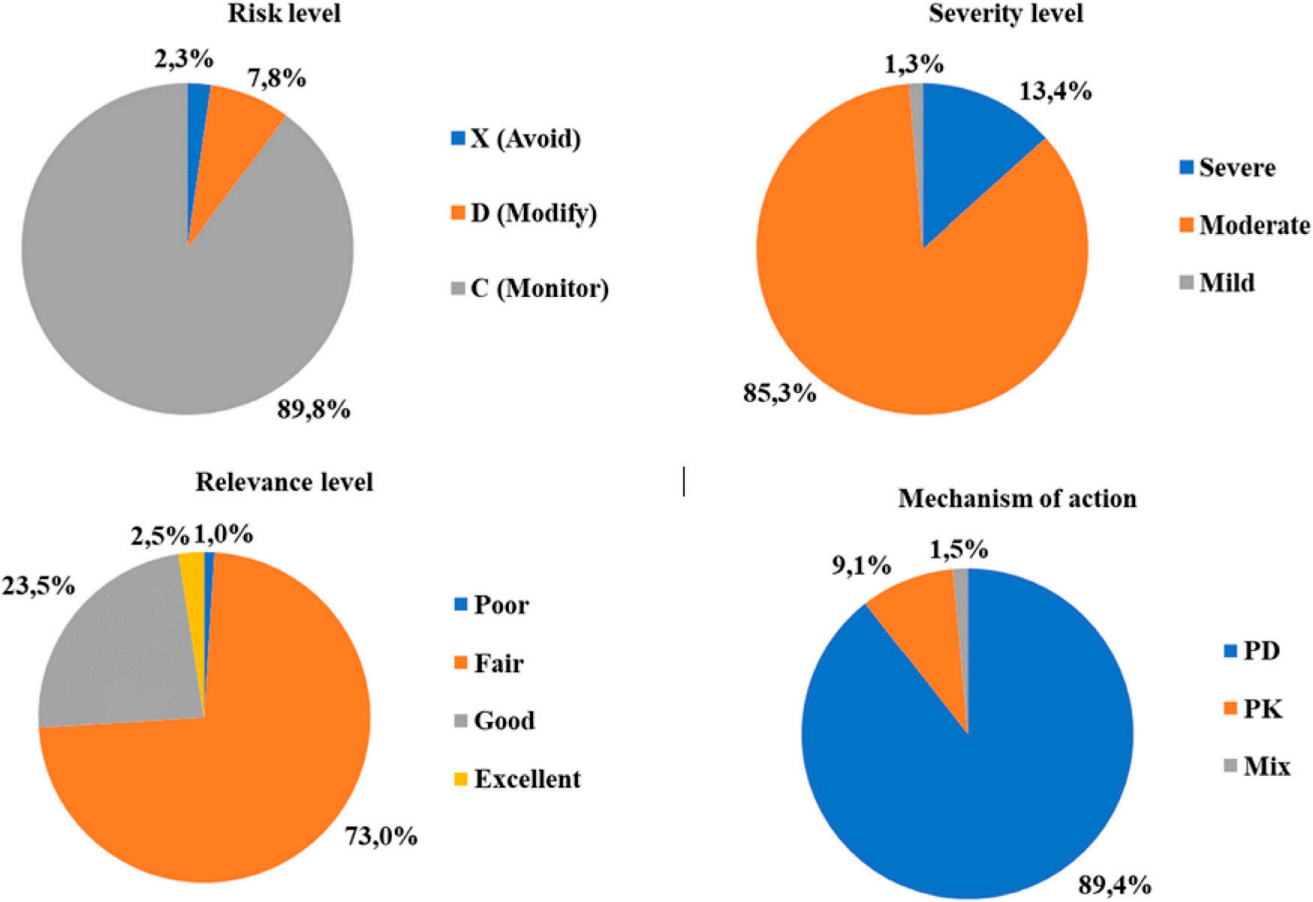
DDI distribution.

It was observed that 89.8% of the described DDIs had a moderate level of severity, 23.5% a good level of relevance, and pharmacodynamic-based DDIs accounted for 89.4% (Figure 1), and 32% of patients had at least one DDI with risk level X or D, indicative of a contraindicated DDI (drug combination is contraindicated or treatment modification should be considered, respectively).

The drugs most frequently involved in potential DDIs detected by the Lexi-Interact^®^ database were quetiapine followed by acetylsalicylic acid, accounting for 24.5% and 10% of the interactions, respectively. In addition, it was detected that antidepressants such as sertraline and trazodone could potentiate the adverse and/or toxic effects of antipsychotics such as quetiapine. The 10 most frequently prescribed drugs are shown in Table 2.

**TABLE 2:** Most frequently prescribed drugs.

Drug	N^a^	ATC code	ATC group
Memantine	39	N06DX	Other anti-dementia drugs
Donepezil	37	N06DA	Anticholinesterases
Acetylsalicylic acid	35	B01AC	Platelet aggregation inhibitors, heparin excl
Omeprazole	34	A02BC	Proton pump inhibitors
Quetiapine	28	N05AH	Diazepines, oxazepines, thiazepines, and oxepines
Atorvastatin	27	C10AA	HMG-CoA reductase inhibitors
Rivastigmine	26	N06DA	Benzodiazepine derivatives
Lorazepam	17	N05BA	Selective β-blocking agents
Bisoprolol	17	C07AB	Other psychostimulants and nootropics
Citicoline	17	N06BX	Anilides

^a^ N, number of times the drug is prescribed in a course of treatment; ATC, anatomical-therapeutic-chemical classification system.

Donepezil and rivastigmine were the AChEIs that interacted most frequently with quetiapine, giving rise to two types of DDIs of moderate severity with a pharmacodynamic mechanism of action. This DDI could lead to higher neurotoxic effect of the antipsychotic drugs, as well as decreased therapeutic effect of the anticholinergic drugs. In addition, the interaction of antidepressants such as sertraline and trazodone with quetiapine could potentiate the adverse and/or toxic effects of antipsychotics such as quetiapine.

Of the 10 most frequent DDI pairs (Table 3), only one, composed of escitalopram and omeprazole, had an excellent pharmacokinetic mechanism of action, excellent relevance, and indicated the possibility that omeprazole could produce an increase in the serum concentration of quetiapine with the consequent risk of adverse events.

**TABLE 3:** Most frequently detected drug pairs with DDIs.

DDI pair	N^a^	ATC code	ATC group
Quetiapine–donepezil	18	N06DA-N05AH	Anticholinesterases: diazepines, oxazepines, thiazepines, thiazepines, and oxepines
Quetiapine–rivastigmine	16	N06DA-N05AH	Anticholinesterases: diazepines, oxazepines, thiazepines, and oxepines
Quetiapine–trazodone	16	N05AH-N06AX	Diazepines, oxazepines, thiazepines, and oxepines—other antidepressants
Bisoprolol–donepezil	12	C07AB-N06DA	Selective β-blocking agents and anticholinesterases
Quetiapine–escitalopram	8	N06AB-N05AH	Selective serotonin reuptake inhibitors: diazepines, oxazepines, thiazepines, and oxepines
Acetylsalicylic acid–metformin	7	B01AC-A10BA	Platelet aggregation inhibitors, heparin excl.—biguanides
Escitalopram–omeprazole	7	N06AB-A02BC	Selective serotonin reuptake inhibitors—proton pump inhibitors—proton pump inhibitors
Quetiapine–lorazepam	6	N05BA-N05AH	Benzodiazepine derivatives: diazepines, oxazepines, thiazepines, and oxepines
Tramadol–trazodone	6	N02AX-N06AX	Other opioids and other antidepressants
Acetylsalicylic acid–sertraline	5	B01AC-N06AB	Platelet aggregation inhibitors, heparin excl.—selective serotonin reuptake inhibitors

^a^ N, number of times the drug is prescribed in a course of treatment.

A total of 97 DDIs were detected between the AChEIs and the remaining drugs administered concomitantly. Donepezil was the drug that presented the highest number of DDIs, with the remaining drugs administered concomitantly (51, 5%). One of the most frequent drug interactions detected was between the AChEIs and beta-blocking agents (n = 29, 4.3%). The prevalence of total and clinically relevant DDIs for severe and moderate severity levels was 17% and 69%, respectively.

The most important patient and/or treatment-related factors that showed the strongest association with the presence of drug interactions were the use of AChEIs (*p* = 0.01) and the total number of drugs (*p* = 0.014) taken by the patient. However, no relationship was observed between the presence of interactions and the number of patient comorbidities (*p* = 0.076), Table 4.

**TABLE 4:** Prevalence of the DDIs according to the factors of the patient.

Variable		DDI’s		*p*
		Yes	No	
Patient gender	Male	36, 8% (32) 63, 2% (55)	30, 8% (4) 69, 2% (9)	0.177
	Female			
Age		80, 6 ± 8,8	77,4 ± 7	0.455
AChEI	Yes	67, 8% (59)	69, 2% (9)	0.01
	No	32, 2% (28)	30, 8% (4)	
Total number of drugs prescribed		8, 2 ± 3, 2	4, 4 ± 1, 7	0.014^a^
Total number of comorbidities		5, 2 ± 2, 6	3, 8 ± 1, 8	0.076^a^

^a^ *Student’s* t*-test*.

## 4 Discussion

This observational study provides information on the epidemiology and potential severity of DDIs in adult patients with dementia. No confounding factors were found to exist between the variables studied (age, gender, total number of drugs prescribed, and total number of comorbidities). The sociodemographic and clinical data of the sample studied are similar to those of other studies, with the mean age range of 80.11–83.2 years (Bogetti-Salazar et al., 2016; Sönnerstam et al., 2018) and with a higher proportion of women, around 58–68.5% (Bogetti-Salazar et al., 2016; Sönnerstam et al., 2018; Oesterhus et al., 2017). However, the percentage of patients affected by AD in our study is higher than that observed in other studies, such as the study by Sönnerstam et al. (2018), where most patients had dementia due to another type of cause. The results highlight the high prevalence of DDIs in the population (87%) and the potential clinical significance of their occurrence in daily clinical practice, both at inpatient (Ruangritchankul et al., 2020; Bogetti-Salazar et al., 2016; Sönnerstam et al., 2018) and outpatient levels (Oesterhus et al., 2017; Trevisan et al., 2021).

In general, DDI prevalence in patients with dementia is high; however, large variability among them (43.2%–76%) has been reported (Ruangritchankul et al., 2020; Bogetti-Salazar et al., 2016; Sönnerstam et al., 2018). However, comparison of the results is complex due to the different scenarios and methodologies, as well as the selection of the reference databases (with diverse severity criteria) and the study designs (Table 5). For instance, a study of potential DDIs in geriatric patients with dementia over 65 years (unlike our study, which included patients under 65 years and with early-onset dementia), used the INTERcheck^®^ database (Trevisan et al., 2021), found a lower prevalence of drug interactions (45%) than that reported in our study (87%) due to differences in the DDI assessment. Conversely, a study to assess the use of psychotropic drugs using the Norwegian Medicine Agency scale (Oesterhus et al., 2017) among outpatients with mild dementia but not including patients in all stages of dementia identified a variety of potentially inappropriate prescriptions and DDIs. One of the most frequent events associated to DDI was “*increased risk of gastrointestinal bleeding*” due to the interaction of acetylsalicylic acid with selective serotonin reuptake inhibitors. In contrast, in our study, we observed more frequent interactions between acetylsalicylic acid and oral antidiabetic drugs, enhancing the hypoglycemic effect of the latter. Similarly, the prevalence of DDIs in elderly patients with dementia in a hospital setting, different to our study, found diverse profiles according with each interactional database used, Micromedex Drug^®^ (Sönnerstam et al., 2018) or Janusmed^®^ (Bogetti-Salazar et al., 2016).

**TABLE 5:** Comparison of drug interaction studies.

Feature	Study			
References	Bogetti-Salazar et al. (2016) *Severe potential drug–drug interactions in older adults with dementia and associated factors*	Oesterhus et al. (2017) *Potentially inappropriate medications and drug–drug interactions in home-dwelling people with mild dementia: drug use in people with mild dementia*	Sönnerstam et al. (2018) *Clinically relevant drug–drug interactions among elderly people with dementia*	Trevisan et al. (2021) *Mild polypharmacy and MCI progression in older adults: the mediation effect of drug–drug interactions*
Study design	Cross-sectional study January 2007–January 2010	Cross-sectional study March 2005–March 2007	Cross-sectional study January 2012 –December 2014	Multi-center population-based cohort
Patients	Outpatients from health institutions in Mexico City	Outpatients from geriatric and psychogeriatric clinics in Norway	Patients from two hospitals in northern Sweden, from the acute internal medicine, orthopedic, and medicines wards	Outpatient from local health unit registers of four Italian cities
Sample	458	251	458	342
Sex (% female)	68.50	58	62.40	61.10
Diagnosis	Dementia	55% AD	Dementia or cognitive impairment	Cognitive status was classified as MCI, CIND, dementia, or preserved cognitive health
N. of drugs	5.2 ± 3.04^a^	4 (2–6)^b^	7.7 ± 3.5^a^	3 (1–4)^b^
Use AChEI	58.60%	42%	No data	No data
Drug interaction database	Micromedex Drug Reax 2.0^®^	Norwegian Medicines Agency, 2014	Janusmed^®^ (formerly Sfinx)	INTERcheck^®^ Computerized Prescription Support System (CPSS)
DDI classification	Two general categories • contraindicated/severe potential DDIs were combined in a *“severe interactions”* group • moderate/minor/absent potential DDIs were combined to form a *“non-severe interaction”* group	Three-point scale • drug interaction only of academic interest • clinicians need to take precautions • drug should not be combined	Four different categories • A to D According to their clinical relevance categories • C and D are considered clinically relevant interactions	DDIs were classified based on their clinical impact into four classes of severity • minor • moderate • major • severe
Number of DDIs	107	191	401	154
DDI mechanism of action	No data	No data	PD: 46.6% PK: 42.1%	No data
Relevance data	Relevance of documenting the most frequent serious and contraindicated DDIs, without indicating their proportion	No data	No data	No data

AD, Alzheimer disease; MCI, mild cognitive impairment; CIND, cognitive impairment no dementia.

^a^ Mean ± standard deviation

^b^ Median and range

The prescription profile coincidently presents similar most prescribed drugs and the number of drugs per treatment. However, the profile of interactions varies considerably according to the database used. The study by Sönnerstam et al. (2018) using the Microdex Drug^®^ database reported 401 DDIs (Sönnerstam et al., 2018) *versus* 107 DDIs found using the Janusmed^®^ database (Bogetti-Salazar et al., 2016).

In our study, the mean number of drugs used per patient was 7.7 ± 3.3, which was similar to the number obtained with the Janusmed^®^ database (Bogetti-Salazar et al., 2016), but it was higher than the data reported using the Micromedex Drug^®^ database (Sönnerstam et al., 2018). Regarding the number of drugs prescribed per patient, the results obtained in the multivariate analysis showed an association between the risk of experiencing a DDI and the number of drugs administered, as in the study by Oesterhus et al. (2017), where the number of drugs was significantly associated with the presence of DDIs.

The Janusmed^®^ interaction database (Bogetti-Salazar et al., 2016), a classification system similar to the Lexi-Interact^®^ database which was used in our study, classifies drug interactions into four different categories according to their clinical relevance, namely, A–D, where categories C and D are considered clinically relevant DDIs. In fact, this database classifies potential DDIs according to the level of risk (e.g., A, B, C, D, and X) and to the level of severity (e.g., mild, moderate, and severe), and considers clinically relevant DDIs those with moderate and severe severity levels. In addition, both databases provide supporting information on the relevance level of the interaction according to the scientific literature and documentation.

In our study, more than one-third of elderly patients with dementia were exposed to greater potential DDIs than that in the study by Sönnerstam et al. (2018), which reported 43.2%, which unlike the study by Bogetti-Salazar et al. (2016) that did not find any anti-dementia drug interactions classified as severe DDIs. In addition, similar to Oesterhus et al. (2017), we found the combination of β-blockers with IAChEs among the most frequent DDIs with the moderate–severity level, which could increase the risk of bradycardia in the patient. From a mechanism point of view, an analysis carried out by the French Pharmacovigilance System (Tavassoli et al., 2007) reported 85% of drug interactions with IAChEs had pharmacodynamic-based mechanisms, accounting for 54.5% of the DDIs.

Potential pharmacokinetic, drug metabolism-based DDIs were identified for donepezil and galantamine as both may undergo CYP2D6 and CYP3A4 metabolism in the liver (Spina et al., 2003). Consequently, their hepatic metabolism may be affected by specific drugs, substrates, inhibitors, or inducers of the same enzymes. A summary of specific drugs in our study that may affect both P450 cytochromes is shown in Table 6. It is worth noting that rivastigmine, another AChEI cognitive enhancer, is less likely to present pharmacokinetic interactions with other drugs as it does not undergo the hepatic metabolism (Monzani et al., 2015). In our study, donepezil and rivastigmine were the AChEIs that showed greater DDI frequency with quetiapine, giving rise to two types of pharmacodynamic-based DDIs of moderate severity, potentially increasing the neurotoxic effect of antipsychotic drugs as well as diminishing the therapeutic effect of anticholinergic drugs.

**TABLE 6:** List of drugs prescribed that are substrates or inhibitors of CYP3A4 and CYP2D6 (Monzani et al., 2015).

Substance name	CYP3A4		Substance name	CYP2D6	
	Substrates	Inhibitors		Substrates	Inhibitors
Amlodipine	+		Amitriptyline	+	
Aripiprazole	+	+	Aripiprazole	+	
Atorvastatin	+	+	Bisoprolol	+	
Bisoprolol	+		Bupropion		+
Bromazepam	+		Carvedilol	+	
Carbamazepine	+		Clomipramine		+
Clomipramine	+		Donepezil		+
Clonazepam	+		Duloxetine	+	+
Clorazepate	+		Fluvastatin		+
Colchicine	+		Galantamine	+	
Diazepam	+		Haloperidol	+	+
Diltiazem	+	+	Levomepromazine	+	+
Domperidone	+		Metoclopramide	+	
Donepezil	+		Mirtazapine	+	
Dutasteride	+		Olanzapine	+	
Escitalopram	+		Omeprazole	+	
Fenofibrate	+		Paroxetine	+	+
Flurazepam	+		Propranolol	+	
Fluvastatin	+		Quetiapine	+	
Haloperidol	+	+	Risperidone	+	
Lansoprazole	+		Sertraline	+	+
Mirtazapine	+	+	Tamsulosin	+	
Nimodipine	+		Tramadol	+	
Nitrendipine	+		Trazodone	+	+
Omeprazole	+		Venlafaxine	+	
Quetiapine	+		Zolpidem	+	
Repaglinide	+				
Risperidone	+				
Sertraline	+	+			
Simvastatin	+	+			
Tamsulosin	+				
Trazodone	+	+			
Venlafaxine	+				
Zolpidem	+				

The study also identified potential DDIs among CNS depressant drugs due to their combined synergistic effect, or with IAChE and serotoninergic drugs with possible increase in the risk of toxicity due to higher antipsychotics effects. Furthermore, multiple interactions of AAS were also found. Sertraline was the SSRI most frequently involved in DDIs with antipsychotics, which could potentiate their adverse and/or toxic effects. In particular, serotonergic drugs may potentiate dopaminergic blockage, increasing the likelihood of neuroleptic malignant syndrome (Berman, 2011).

Unlike other studies (Ruangritchankul et al., 2020; Bogetti-Salazar et al., 2016; Oesterhus et al., 2017; Trevisan et al., 2021), we addressed the type of interaction according to its possible mechanism and noticed that DDIs with a pharmacodynamic-based mechanism generally predominated.

A factor to take into account is the fact that the evidence and/or relevance score supporting the DDIs detected according to the Lexi-Interact^®^ database is “Fair” in 73% of the treatments analyzed and “Good” in 23.5%. This adds value to the evaluation of the DDI, which usually is not reported in DDI studies, with the exception of the study by Bogetti-Salazar et al. (2016), which highlights the relevance of documenting the most frequent, serious, and contraindicated DDI; however, in their study, they do not provide information on what proportion of the interactions detected present a good level of clinical evidence.

## 5 Limitations

One of the limitations of this study is that it is limited to a specific population. It would be necessary to increase the study population in order to make the results obtained more representative. It would be necessary in future studies to expand the population of dementia patients to include patients from different geographical areas or of different socio-economy to make the results more generalizable. On the other hand, the cross-sectional design is another limitation of the study as it is able to assess long-term outcomes of DDIs. When the results are obtained, we consider that a longitudinal study is necessary, and we are in the process of setting up additional studies to address longitudinal evaluation of the patients to provide insights into the long-term impacts of these interactions on patient health and hospitalization rates. In addition, to confirm that the interactions detected are representative, future studies should be analyzed by increasing the number of databases.

## 6 Conclusion

In conclusion, the high number of DDIs present in patients with dementia is observed in this study. A total of 89.8% DDIs present a moderate level of severity, which is a potential risk for the occurrence of adverse reactions. In addition, we found that the presence of AChEIs in drug treatments is a potential factor in the occurrence of DDIs, being involved in a high number of clinically relevant DDIs, especially with antipsychotics, such as quetiapine. These DDIs may result in an increased risk of neurotoxic adverse effects. The PD mechanism of action could explain 89.4% of the DDI found in the present study. The most frequent PD interaction was the interaction between AChEIs and bradycardic drugs, especially beta-blocking agents. These complex DDIs add considerable challenges to the treatment of patients with dementia as they may not only compromise therapeutic efficacy but also increase the risk of serious adverse events. This fact highlights the need for more accurate tools to assess the severity and potential impact of DDIs in clinical practice.

The results highlight the difficulty of assessing DDIs in clinical practice, in terms of potential severity and impact on therapy, as well as the need to use and compare different databases for decision-making. Although there are few studies of drug interactions in patients with dementia, it is important to pay attention when prescribing new drugs to these patients. This should be encouraged with the creation of multidisciplinary groups in which health professionals perform a joint and comprehensive review of their treatments, taking into account factors such as the administration of drugs that inhibit or induce hepatic metabolism or P450 substrate overlapping. There would be several approaches and actions that could improve the outcomes derived from the DDI. Some actions may include de-prescription programs, medication review on regular basis, dosing adjustment, TDM when necessary, and review of signs and symptoms in the patients for adverse effects. Finally, although this study provides a solid basis for understanding DDIs in patients with dementia, further research is needed to address gaps in the current literature. Larger studies are needed to explore the long-term implications of DDIs in this population and to evaluate strategies that minimize the risk of adverse interactions, thereby improving the safety and efficacy of drug treatment in patients with dementia.

## Data Availability

The original contributions presented in the study are included in the article/Supplementary Material; further inquiries can be directed to the corresponding author.
